# The prognosis of iatrogenic saphenous nerve injuries during hamstring tendon harvesting in anterior cruciate ligament reconstruction

**DOI:** 10.1186/s13018-024-04929-z

**Published:** 2024-07-24

**Authors:** Omer Faruk Egerci, Fırat Dogruoz, Mehmet Melih Asoglu, Mehmet Barıs Ertan, Aliekber Yapar, Ozkan Kose

**Affiliations:** Department of Orthopedics and Traumatology, University of Health Sciences, Antalya Training and Research Hospital, Varlık mah., Kazım Karabekir cd, Muratpasa, Antalya, 07100 Turkey

## Abstract

**Purpose:**

This study aims to evaluate the long-term outcomes of saphenous nerve (SN) injuries from hamstring tendon harvesting during ACL reconstruction, focusing on clinical results and patient satisfaction after at least two years. Additionally, it investigates the incidence, recovery patterns, and impact of these injuries on functional outcomes, daily activities, and ACL re-rupture rates immediately post-surgery and at final follow-up.

**Materials and methods:**

A retrospective review was conducted on patients who had undergone ACL reconstruction with hamstring tendon grafts at a single institution between January 2015 and January 2020. The incidence of SN injuries was assessed immediately after surgery and at final follow-up. Additionally, the recovery rate and time were evaluated, and the impact of these injuries on functional outcomes was measured using the Lysholm Knee Score (LKS) and patient-reported effects on daily activities.

**Results:**

Of the 159 patients analyzed, iatrogenic SN injuries were initially observed in 87 (54.7%) patients post-ACLR. By the final follow-up, paresthesia had resolved in 36 (22.6%) patients within an average of 11.1 months. Persistent SN injuries were recorded in 51 (32.1%) patients, affecting various extents of the infrapatellar branch (IPBSN) and the sartorial branch (SBSN) of the saphenous nerve. Patients with persistent SN injuries experienced a significant impact on daily activities and had lower LKS scores compared to those without injuries or with recovered injuries. Furthermore, a higher re-rupture rate was associated with persistent SN injuries.

**Conclusions:**

The study finds that SN injuries during hamstring graft harvesting for ACL reconstruction are common, with a significant portion of patients experiencing persistent sensory deficits for at least two years postoperatively. These injuries are observed to adversely affect patient satisfaction and functional outcomes and to increase the re-rupture rate.

## Introduction

Arthroscopic anterior cruciate ligament reconstruction (ACLR) is a well-established orthopedic procedure for symptomatic patients with ACL ruptures. The goal of managing ACL insufficiency is to restore knee function, primarily to allow patients to return to pre-injury activity levels [1, 2]. This can potentially prevent the onset and progression of knee osteoarthritis, achieved through early surgery and postoperative rehabilitation essential for restoring knee joint stability and function [3, 4]. Many ACL reconstruction techniques utilize autografts due to their effectiveness in restoring joint stability [5]. Various autologous graft options are available, including bone-patellar tendon-bone, hamstring tendons (HT), peroneus longus tendon, and the quadriceps tendon. Each grafting option has its own advantages and disadvantages, making it challenging to reach a consensus on the optimal graft option [6–9]. However, hamstring tendons are the most commonly used autografts for ACLR [9] due to their ease of harvesting, superior biomechanical strength compared to the native ACL, straightforward preparation, and reliable fixation methods [6–10].

After passing through the adductor canal, the saphenous nerve, a terminal cutaneous branch of the femoral nerve, divides into two main branches: the infrapatellar branch and the sartorial branch. The infrapatellar branch (IPBSN) extends medially to the knee, providing sensory innervation to the anteromedial aspect of the knee. The sartorial branch (SBSN) runs alongside the great saphenous vein, innervating the medial part of the leg down to the ankle [11–13]. Due to their close proximity to the surgical field, iatrogenic injuries to these nerve branches may occur during tendon harvesting, anteromedial portal placement, and tibial tunnel drilling, particularly when harvesting hamstring tendons [14–16]. The incidence of sensory nerve (SN) injuries after ACLR with HT grafts is highly variable, reported to range from 0 to 88% in the literature [17, 18].

The saphenous nerve is purely sensory; thus, functional outcomes are generally not affected by the loss of sensation, except in terms of reduced patient satisfaction [19–23]. Despite this, the long-term prognosis of these injuries is poorly documented [19, 24], and the extent of recovery over time remains unclear. This lack of detailed understanding prompts a need for more focused research, particularly regarding the impact of SN injuries on long-term functional outcomes. This study hypothesizes that saphenous nerve (SN) injuries during hamstring tendon harvesting for anterior cruciate ligament (ACL) reconstruction may decrease patient satisfaction and functional results. The primary aim of this study is to evaluate the long-term prognosis of iatrogenic saphenous nerve (SN) injuries incurred during hamstring tendon harvesting for anterior cruciate ligament (ACL) reconstruction and to assess their impact on clinical outcomes and patient satisfaction at a minimum of two years postoperatively. Additionally, the secondary aim is to determine the incidence, recovery rate, and patterns of SN injuries immediately after surgery and at the final follow-up. This study will also investigate the effects of these injuries on functional outcomes, patient-reported impact on daily activities, and their relationship with ACL re-rupture rates.

## Materials and methods

### Patients and study design

**Fig. 1:**
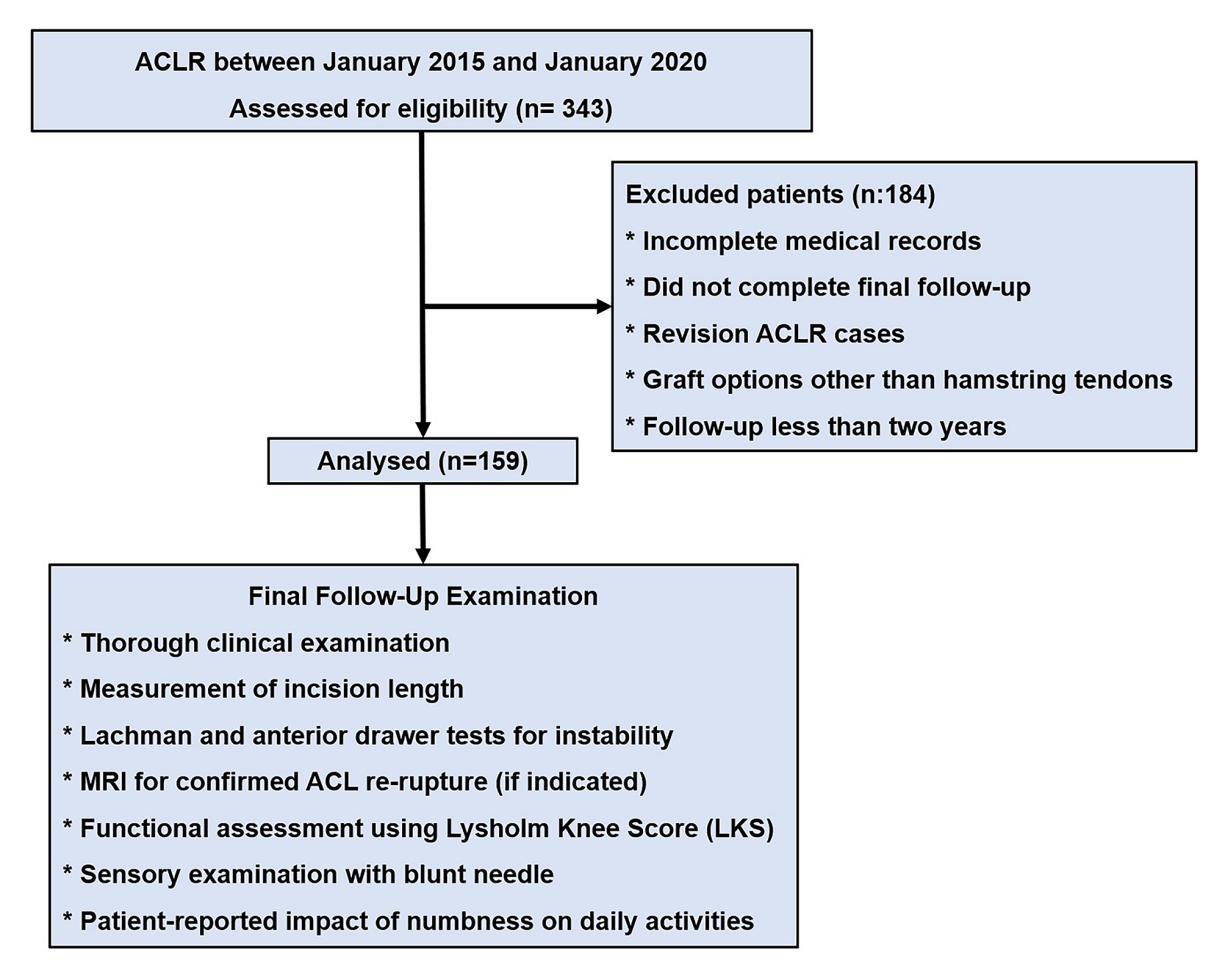
Flowchart of Patient Selection and Follow-Up in ACLR Study

A retrospective review of digital medical records was conducted to identify all patients who underwent ACLR using a hamstring tendon graft at our institution between January 2015 and January 2020. Patient charts, operation notes, medical records, and notes taken during follow-up visits were collected from the institutional patient database. Patients with incomplete medical records, those who did not complete the final follow-up, revision ACLR cases, and those who underwent ACLR with grafts other than hamstring tendons were excluded from the study. Additionally, patients with less than two years of follow-up were excluded (Fig. 1). The research was conducted by the ethical principles outlined in the 1964 Helsinki Declaration and its subsequent revisions. The institutional review board approved the study protocol (Approval date/issue: 22.12.2020/20.06–390).

### Hamstring tendon harvesting technique

In all cases, ACLR was performed using a thigh tourniquet under spinal anesthesia. After confirming ACL rupture through diagnostic arthroscopy, the hamstring tendons, specifically the Gracilis and Semitendinosus (ST) tendons, were harvested. A 4–5 cm oblique incision was made, the sartorial fascia was cut following soft tissue dissection, and the Gracilis and Semitendinosus (ST) tendons were identified, sutured, released from their attachments, and harvested with a closed tendon stripper. Anatomic graft placement was achieved by drilling the femoral tunnel through the anteromedial portal and the tibial tunnel using a 55-degree tibial guide. The graft was secured with an EndoButton on the femoral side, a bioabsorbable interference screw, and a titanium U staple on the tibial side.

### Clinical assessments

Patients were assessed at two main time points: immediately after anterior cruciate ligament reconstruction (ACLR) to observe the initial incidence of saphenous nerve (SN) injuries, and at the final follow-up (mean 58.9 months postoperatively, range 25–92 months) to evaluate recovery rates and the ongoing impact of SN injuries on functional outcomes and patient satisfaction. A thorough clinical examination was performed on all patients at the final follow-up. The length of the incision was measured from the incision scar. Instability was evaluated using the Lachman and anterior drawer tests. In cases where patients presented with positive clinical findings and complaints of instability, an MRI was performed to confirm ACL re-rupture. Functional outcomes were assessed using the Lysholm Knee Score (LKS), which was graded as follows: 95–100 points (‘excellent’), 84–94 points (‘good’), 65–83 points (‘fair’), and ≤ 64 points (‘poor’) [25–27]. The sensory examination involved a light touch test over the SN dermatome with a blunt needle. Patients experiencing postoperative paresthesia were asked about the impact of numbness on their daily activities. The degree of numbness was categorized by the patients as none, mild, moderate, or severe.

### Statistical analysis

Descriptive statistics for categorical variables were presented as frequencies and percentages, and continuous variables were presented as mean ± standard deviation and range. The Kolmogorov-Smirnov test was used to test normality. Comparative analysis between independent groups was performed using the ANOVA and chi-square tests. A value of *p* < 0.05 was accepted as statistically significant.

## Results

**Fig. 2:**
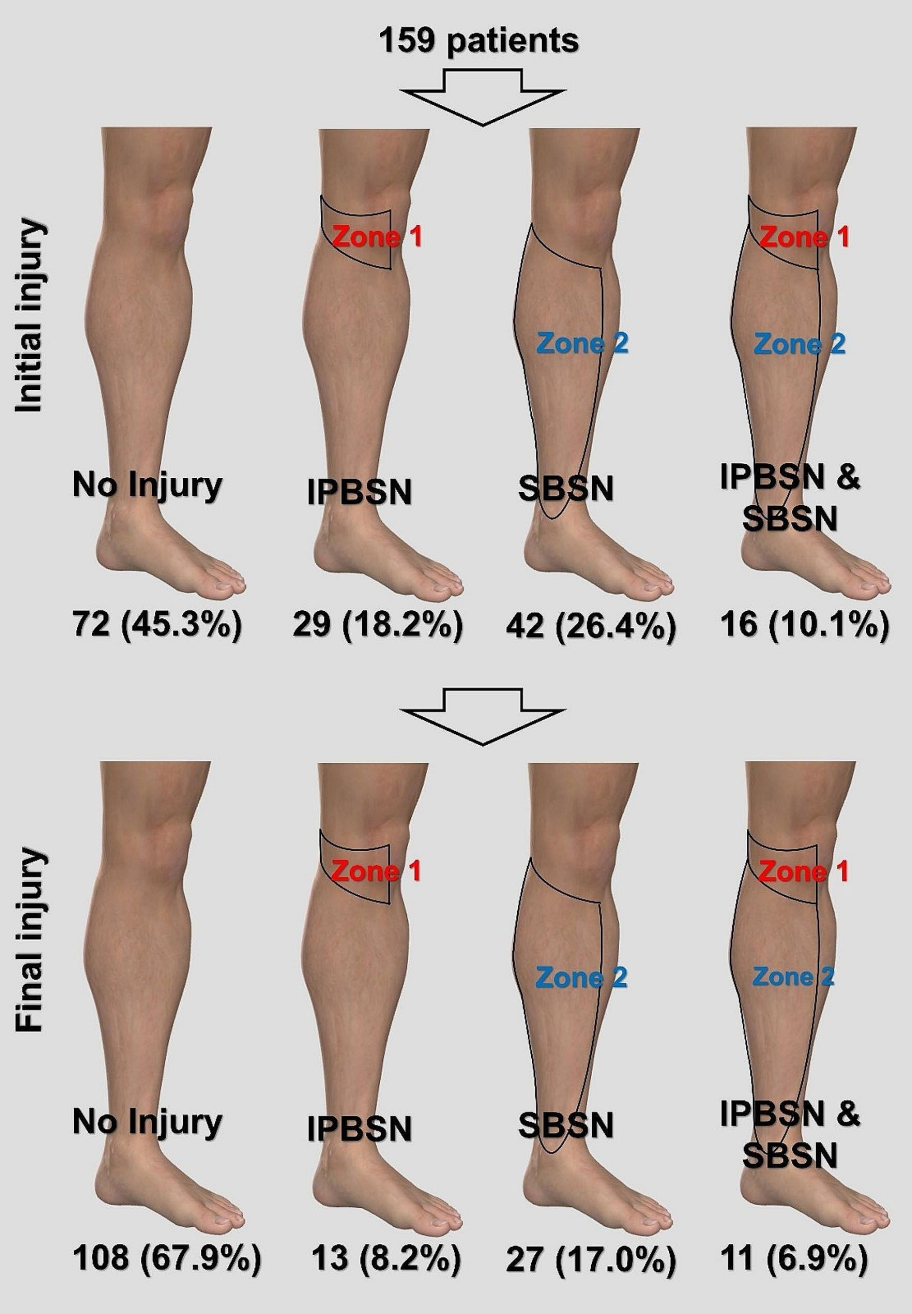
Illustration showing the rate of initial SN injury and the final SN injury in the study population

A total of 159 patients (16 female, 143 male) with a mean age of 33.8 ± 8.9 years (range 19–54 years) were included in the study. The right knee was affected in 75 (47.2%) patients, while the left knee was affected in 84 (52.8%). The mean follow-up duration was 58.9 ± 16.7 months (range 25–92 months). Iatrogenic SN injury was observed in 87 (54.7%) patients immediately after ACLR. By the final follow-up, paresthesia had resolved in 36 (22.6%) patients within an average of 11.1 ± 7.4 months (range 1–24 months). SN injury remained consistent for at least two years in 51 (32.1%) patients. Among these patients, involvement of the isolated IPBSN dermatome (Zone 1) was noted in 29 (18.2%), the isolated SBSN dermatome (Zone 2) in 42 (26.4%), and both dermatomes in 16 (10.1%) (Fig. 2). Recovery rates were similar across SN injury sites (Zone 1: 55.2%, Zone 2: 35.7%, Combined: 31.3%; p = n.s).

Out of the 87 patients with initial nerve injury, 6.9% reported a severe impact, 30.8% a moderate impact, and 17.0% no impact on their daily life due to paresthesia. Accompanying meniscal injuries (lateral, medial, or combined) were present in 67.3% of patients and were treated either with partial meniscectomy or meniscal repair. The incidence of meniscal injuries was comparable among the persistent, recovered, and no injury groups (p = n.s).

Of the 159 patients, 19 (11.9%) exhibited clinical findings consistent with re-rupture and were excluded due to low LKS secondary to re-rupture to avoid bias. The remaining 140 patients with intact grafts were analyzed to determine the impact of paresthesia on functional outcomes. Of these, 68 (48.5%) had no SN injury, 34 (24.2%) recovered from SN injury, and 38 (27.1%) had persistent SN injury. LKS scores were similar between the uninjured and recovered groups, both demonstrating significantly higher scores than the group with persistent injury (Table 1). Demographic and clinical characteristics were similar among the three groups. A significantly higher re-rupture rate was observed in patients with persistent SN injury (*p* = 0.001) (Table 2).

**Table 1:** Comparison of clinical and functional results of iatrogenic SN injury among intact ACL graft

Variables	Uninjured (*n*:68)	Recovered (*n*:34)	Consistent (*n*:38)	*p*
Age (year ± SD)	33.9 ± 8.8	34.0 ± 8.8	34.1 ± 9.7	n.s.
Sex (Men)	64 (94.1%)	30 (88.2%)	33 (86.8%)	n.s.
Side (Right)	30 (44.1%)	20 (58.8%)	17 (44.7%)	n.s.
Follow-up (months ± SD)	59.6 ± 16.9	61.2 ± 16.9	58.1 ± 16.7	n.s.
Incision Length (cm ± SD)	4.8 ± 0.9	5.0 ± 1.0	4.6 ± 0.8	n.s.
Zone	-			n.s.
*IPBSN injury*		14	7	
*SBSN injury*		15	22	
*Combined injury*		5	9	
LKS (score ± SD)	96.8 ± 5.9^a^	95.0 ± 6.4^b^	90.6 ± 9.1^c^	0.001^1^
*Excellent*	53	24	15	0.006
*Good*	10	6	14	
*Fair*	5	4	8	
*Poor*	0	0	1	

*Abbreviations*,* SD: Standard deviation*,* LKS: Lysholm Knee Score*

^1^ ANOVA Post-hoc Bonferroni adjustment. a vs. b p = n.s. a vs. c *p* = 0.001 b vs. c *p* = 0.031

**Table 2:** Comparison of re-rupture according to SN injury at the final follow-up

ACL graft continuity	SN injury at the final follow-up			*p-value*
	**No**	**Yes**	**Total**	
Intact ACL graft	102 (72.9%)	38 (27.1%)	140	0.001^1^
Re-ruptured ACL graft	6 (31.6%)	13 (68.4%)	19	
Total	108 (67.9%)	51 (32.1%)	159	

*Abbreviations: ACL: anterior cruciate ligament*

^1^ Chi-Square test

## Discussion

This study demonstrated that iatrogenic saphenous nerve (SN) injury was a common complication following ACLR with hamstring tendon autografts, occurring in over half of patients initially. Although a substantial proportion of patients experienced resolution of paresthesia within an average of 11.1 months, a significant number (32.1%) continued to have persistent SN injury at two years post-operatively. These persistent injuries significantly affected patient outcomes, with those experiencing unresolved SN injuries reporting substantially lower Lysholm Knee Scores compared to those without injuries or with resolved injuries. This underscored the profound impact of these nerve injuries on long-term patient satisfaction and functional performance. Notably, the recovery rate was not influenced by the specific site of SN injury.

According to the current study, iatrogenic SN injuries were observed in nearly half of the patients (54.7%) despite using an oblique incision. However, by the end of at least two years of follow-up, approximately 40% of those who initially sustained SN injuries had recovered, reducing the overall rate of persistent SN injuries to 32.1%. This suggests that some injuries may be temporary and likely represent neuropraxia. The average recovery period was observed to be one year, although recovery could take up to two years for some patients.

Although the rate of SN injuries at specific follow-up visits is reported in many studies, consecutive follow-ups and recovery are documented in very few [13, 19, 22–24, 28–31]. Comparing the data is challenging because follow-up times vary, and different incision and harvesting techniques are employed. Recovery rates are reported to range from 0 to 92% at various time points. It is suggested that recovery rates might increase over time; however, the literature presents contradictory data. A recovery rate of 65% at 6 months was reported by Sipahioglu et al. [28], whereas a recovery of 12.7% at an average follow-up of 32 months was reported by Mochizuki et al. [24]. Additionally, Papastergiou et al. [32] proposed that hypoesthesia remains permanent after one year; however, in the current study, recovery continued for up to two years. In light of the findings from previous studies and this study, predicting the timing of recovery is challenging. Reports of complete recovery are accompanied by studies documenting partial improvements and reductions in the area of sensory loss. It was reported by Mahmood et al. [22] that the area of numbness decreased from 43.4 cm^2 to 37.9 cm^2 within six months. Similarly, a significant narrowing of the numbness area at repeated follow-ups was demonstrated by Sipahioglu et al. [28] and Kjærgaard et al. [17].

Building upon previous research, the current study further revealed that both terminal branches of the saphenous nerve (SN)—the infrapatellar branch (IPBSN) and the sartorial branch (SBSN)—can be injured, either independently or in combination. This finding suggests that the injury site is not always directly correlated with the surgical dissection at the skin incision but may occur during tendon harvesting with the tendon stripper or during dissection of interconnections between hamstring tendons. While several studies in the literature have reported only IPBSN injuries, with no mention of SBSN injuries [17, 20–24, 28, 31–37], others have differentiated between the two but did not report combined injuries. A few studies, however, have documented both isolated and combined injuries.

In two previous studies with the largest number of patients examined, only IPBSN injury following HT graft harvesting was reported by both Papastergiou et al. [32] (230 patients) and Ochiai et al. [21] (123 patients). In contrast, Sharaby et al. explored the effects of vertical and oblique incisions on saphenous nerve (SN) injuries and identified 39 cases of SN injuries in a cohort of 84 patients. They noted that most of the sensory loss was localized to the IPBSN in 27 patients (69.2%), while the sartorial branch (SBSN) was affected in only 12 patients (30.8%), with no instances of combined injuries reported [19]. Whereas, a rate of 74% SN injuries with a vertical incision was reported by Sanders et al., with 14 (23%) having isolated SBSN injury, 12 (19%) having isolated IPBSN injury, and 20 (32%) having combined SBSN and IPBSN injuries [13].Considering the information in their anatomical dissection study and observed injury patterns, it was suggested that the IPBSN injury was closely related to the skin incision, but the tendon stripper was the cause of injury to the SBSN. Compared to the data in these studies, more isolated or combined total SBSN injuries were observed in our study. It is thought that the conflicting rates of injury reported in different studies are related to the variation of SN branches and tendon harvesting techniques.

While it has generally been reported that SN injuries do not affect clinical outcomes, they do decrease patient satisfaction [17, 21, 23, 28, 31]. Indeed, Musevi et al. noted that many patients were unaware of their numbness until it was clinically assessed [34], and Keyhani et al. observed that patients often disregard this condition [35]. In the present study, of the 87 patients with SN injuries, severe impact was reported by 6.9%, moderate impact by 30.8%, and no impact on daily life due to paresthesia by 17.0%. Moreover, LKS was found to be lower in patients with persistent numbness at the last follow-up visit compared to patients with no injury and those who had recovered. In addition, a significantly higher rate of re-rupture was found in patients with persistent SN. Dysesthesia, hypoesthesia, neuroma, reflex sympathetic dystrophy, anterior knee pain, and kneeling pain can all be caused by saphenous nerve injury. Patient satisfaction and quality of daily life can be negatively affected by these complications [20, 38]. Pain due to neuroma and instability due to loss of proprioception may occur after saphenous nerve injury in these patients [39, 40]. Therefore, full recovery of knee stability requires both surgical reconstruction and the restoration of proprioceptive control [41]. This is further supported by the fact that a significant proportion of patients with re-rupture in present study had SN injuries. Although SN injury is often described as a minor complication, the high rate of re-rupture observed suggests that it may not only induce hypoesthesia but also contribute to re-rupture as a major complication. However, this conclusion needs to be further supported by studies with a larger number of patients. Additionally, considering the multifactorial nature of re-rupture, other potential factors should be examined in detail.

Although the present study utilized an oblique incision, in line with recommendations aimed at minimizing the risk of saphenous nerve (SN) injuries [38, 40, 42–47], the incidence of such injuries remained notably high. This suggests that incision type alone may not fully protect against SN injury, as other factors likely contribute. While studies show oblique or horizontal incisions are generally safest as they follow the SN nerve’s natural path [40, 42, 43], and vertical incisions are discouraged due to increased risk [38, 45–47], differences in surgical techniques, anatomical variations among patients, and surgeon expertise can also influence outcomes [13, 48, 49]. Consequently, a multi-faceted approach is essential, incorporating meticulous surgical techniques, strategic patient positioning (such as the figure-of-four position) [48], and possibly alternative harvesting methods (e.g., employing a posteromedial or popliteal approach, or isolating the semitendinosus tendon for grafting) [18, 31, 50, 51]. These strategies could further diminish the occurrence of SN injuries and improve overall surgical outcomes.

Strengths of this study include a greater number of patients and a longer follow-up period than most similar research in the literature, which enhance its ability to track recovery effectively. Furthermore, it comprehensively analyzes all terminal branch injuries. Despite these strengths, this work is not without limitations; its retrospective design and reliance on patient self-reports for postoperative data could introduce recall bias. Nonetheless, patients underwent detailed examinations during the last follow-up visit, mitigating some concerns related to data accuracy.

The study concludes that iatrogenic saphenous nerve (SN) injuries are common after ACL reconstruction using hamstring tendon autografts, affecting over half of the patients initially. Long-term, these injuries are linked to lower patient satisfaction and worse functional outcomes. These persistent injuries significantly impact quality of life and functional performance, underscoring the need for improved surgical techniques and post-operative management.

## Data Availability

No datasets were generated or analysed during the current study.
